# Viral Metagenomic Analysis of *Aedes albopictus* Mosquitos from Southern Switzerland

**DOI:** 10.3390/v12090929

**Published:** 2020-08-24

**Authors:** Jakub Kubacki, Eleonora Flacio, Weihong Qi, Valeria Guidi, Mauro Tonolla, Cornel Fraefel

**Affiliations:** 1 Institute of Virology, University of Zürich, CH-8057 Zürich, Switzerland; 2 Laboratory of Applied Microbiology, Department for Environment Constructions and Design, University of Applied Sciences and Arts of Southern Switzerland, CH-6500 Manno, Switzerland; eleonora.flacio@supsi.ch (E.F.); valeria.guidi@supsi.ch (V.G.); mauro.tonolla@supsi.ch (M.T.); 3 Functional Genomics Center Zurich, CH-8057 Zürich, Switzerland; weihong.qi@fgcz.ethz.ch

**Keywords:** metagenomic, *Aedes albopictus*, virome

## Abstract

A metagenomic study was performed on 498 female and 40 male *Aedes albopictus* mosquitos collected in August and September 2019 in Ticino, a region in southern Switzerland, to address the question regarding the risk of the local transmission of zoonotic viruses. A total of 13 viruses from seven different virus families and several unclassified viral taxa were identified. Reads of insect-specific flaviviruses were present in all pools, and a complete genome of aedes flavivirus was assembled and phylogenetically analysed. The most abundant virus was Wenzhou sobemo-like virus, assembled from 1.3 × 10^5^ to 3.6 × 10^6^ reads in each pool. In a pool of male mosquitos, a complete genome of aedes Iflavi-like virus was detected and phylogenetically analysed. Most importantly, genomes of human pathogenic viruses were not found. This is the first study to determine the virome of *Ae. albopictus* from Switzerland and forms a baseline for future longitudinal investigations concerning the potential role of *Ae. albopictus* as a vector of clinically relevant viruses.

## 1. Introduction

The Asian tiger mosquito *Aedes albopictus* plays an important role as a vector of arboviruses, many of which can severely affect human health, including dengue virus (DENV), Zika virus (ZIKV), yellow fever virus (YFV), and chikungunya virus (CHIKV) [1]. Changing environmental factors and progressing human development can support the establishment of invasive arthropod species, affect the vector competence of endemic species, and enhance viral virulence, thereby potentially contributing to the spread of emerging viral diseases. *Ae. albopictus*, originally endemic in South-East Asia, has spread over the past approx. 40 years throughout large parts of the Americas, Africa, Australia, and Southern Europe [2]. In Switzerland, *Ae. albopictus* was spotted for the first time in 2003 in canton Ticino, where since 2007 it has been firmly established; occasionally it is also found in northern parts of Switzerland [3,4,5,6,7]. To date, no autochthonous cases of DENV or CHIKV infections have occurred in Switzerland, while in neighbouring countries, i.e., Italy and France, several cases have been reported. The first transmission of CHIKV by *Ae. albopictus* outside of a tropical area was reported in 2007 in Italy [8]. Several cases of autochthonous DENV and CHIKV virus infections have been linked to *Ae. albopictus* as a vector in France as well [9,10]. Hence, *Ae. albopictus* appears to retain its competence to harbour and transmit zoonotic viruses outside of the tropical regions.

In addition to zoonotic viruses, mosquitos are known to host many different insect-specific viruses (ISVs) which persistently infect mosquitos, but not vertebrates [11], although many ISVs are closely related to human pathogens [12]. Interestingly, coinfection with some ISVs may modulate or even supress replication of specific zoonotic viruses, such as West Nile virus (WNV), in mosquito cells and therefore inhibit their transmission [13,14,15,16]. In addition, coinfection with specific bacteria such as *Wolbachia* sp. may limit the ability of mosquitos to transmit specific zoonotic viruses [17].

As *Ae. albopictus* is endemic in Ticino and progressively invades other regions of Switzerland, and because the presence of the vector facilitates the possibility that zoonotic viruses may be locally transmitted to humans, we addressed the question as to whether these mosquitos indeed harbor such viruses. Specifically, using next-generation sequencing and metagenomic analyses, we determined the full, unbiased virus population diversity of *Ae. albopictus* collected in five municipalities of Lugano in Ticino. A similar study has not previously been performed in Switzerland.

## 2. Materials and Methods

### 2.1. Sample Collection

**Figure 1:**
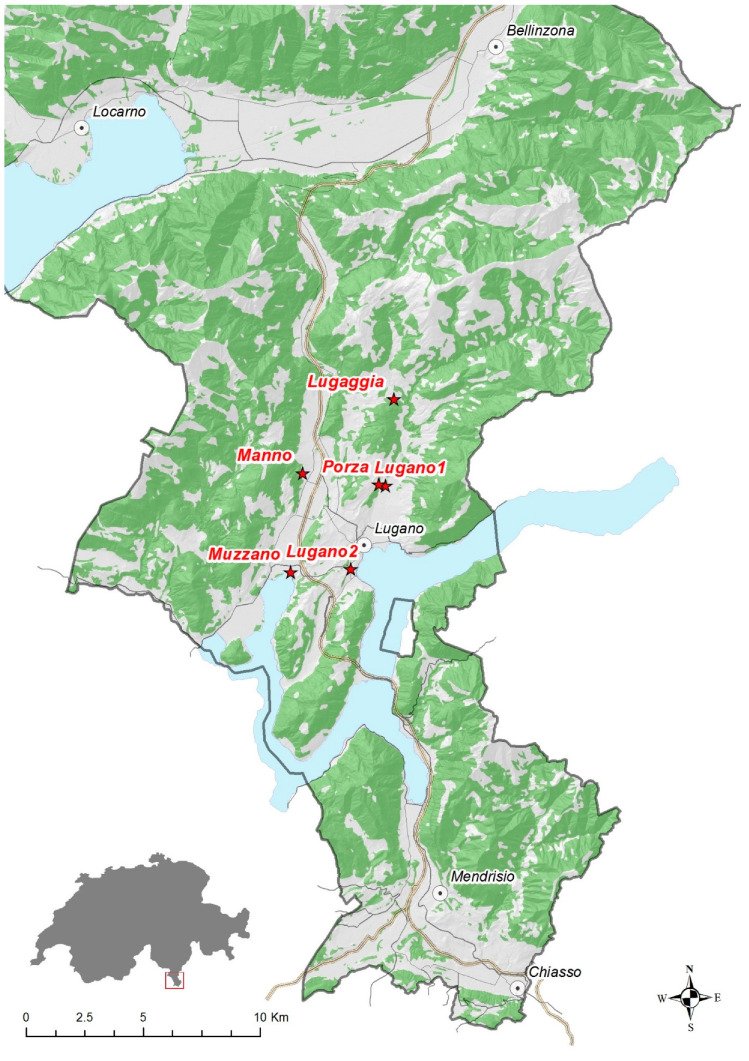
Locations of mosquito collection. Detailed coordinates and sample descriptions are given in Table 1.

Adult mosquitos were captured in urban and public areas, using electric manual aspirators and entomological nets in August and September 2019 in five municipalities of the Lugano area (Lugaggia, Manno, Porza, Muzzano, and Lugano), canton Ticino, Switzerland (Figure 1). Mosquitos were euthanised by exposure to dry ice [18] and identified to the species level using morphological keys [19,20,21,22,23]. In total, 538 adults (males *n* = 40; females *n* = 498) were collected, divided into 15 pools according to collection date, place, and gender (5–59 mosquitos per pool; see Table 1), and stored at −20 °C until further use.

### 2.2. Sample Processing and Sequencing

To each pool, 500 µL of phosphate-buffered saline (PBS) (Merck, Darmstadt, Germany) and a stainless-steel bead (5 mm, Qiagen, Hilde, Germany) were added, and samples were mechanically homogenised in a TissueLyser II (Qiagen, Hilde, Germany) at 20 Hz for 2 min. Then, homogenates were centrifuged at 16,060× *g* for 5 min, and the supernatants were passed through a 0.45 µm syringe filter (Puradisc, 13 mm, Whatman GE Healthcare, Chicago, IL, USA). To enrich nucleic acids that are protected by a virus capsid, 14 µL of micrococcal nuclease buffer, 1 µL of micrococcal nuclease (both New England Biolabs, Ipswich, MA, USA), and 1 µL of ribonuclease A from bovine pancreas (Merck, Darmstadt, Germany) were added to 134 µL of each filtrate from the previous step and incubated for 15 min at 45 °C and 1 h at 37 °C. Total RNA and DNA were extracted using the QIAmp Viral RNA mini kit (Qiagen, Darmstadt, Germany) without RNA carrier. β-mercaptoethanol (Bio-rad, Cressier, Switzerland) was added at a final concentration of 1% in order to inactivate nucleases. RNA was transcribed using 2.5 µM of random primer with a known 20 nucleotide (nt) tag sequence at the 5′ end (SISPA-N: GTTGGAGCTCTGCAGTCATCNNNNNN) and the RevertAid First Strand H minus cDNA Synthesis Kit (Thermo Fisher Scientific, Basel, Switzerland) following the manufacturer’s recommended protocol. Then, 1 µL of RNase H (New England Biolabs, Ipswich, MA, USA) was added to degrade remaining RNA. A premix of 0.8 µM SISPA-N primer, 10× Klenow buffer, and 0.2 mM dNTP was added to 45.5 µL of the first-strand DNA. Following denaturation at 95 °C for 1 min and cooling down on ice, the second strand was synthesized using Klenow polymerase (5 U/20 µL; Thermo Fisher Scientific, Basel, Switzerland) for 15 min at 25 °C followed by 1 h at 37 °C. An additional step of second-strand synthesis using Klenow polymerase was performed at the same conditions, followed by DNA purification using the PureLink^®^ PCR Micro Kit (Invitrogen-ThermoFisher, Waltham, MA, USA). Then, dsDNA was amplified non-specifically by sequence-independent single primer amplification (SISPA). For this, the HotStarTaq DNA polymerase (Qiagen, Darmstadt, Germany) and the SISPA primer (GTT GGA GCT CTG CAG TCA TC) were used under the following conditions: 15 min of activation at 95 °C, 18 cycles of 30 s at 94 °C, 30 s at 58 °C and 1 min at 72 °C, followed by 10 min at 72 °C and cooling down to 4 °C. Finally, the amplified products were purified using the QIAquick PCR purification kit (Qiagen, Darmstadt, Germany). The total DNA was quantified on the Agilent 4200 TapeStation (Santa Clara, CA, USA). Sequencing libraries were made using NEBNext Ultra II DNA library prep kit and NEBNext^®^ Multiplex Oligos for Illumina^®^ (96 Unique Dual Index Primer Pairs) (both New England Biolabs, Ipswich, MA, USA). After quantifying at the TapeStation and pooling at equal molar concentrations, libraries were sequenced using the Illumina NovaSeq system in a single-end 1 × 100 nt run at the Functional Genomics Center Zurich (FGCZ, Zurich, Switzerland).

### 2.3. Quality Control, Pre-Processing, and Assembly of Metagenomics Reads

Individual metagenomes per sample and the metagenome of all samples combined were assembled and analyzed using the same pipeline. In detail, the technical quality of Illumina single-end (SE) DNA-seq reads was evaluated using FastQC version 0.11.7 (https://www.bioinformatics.babraham.ac.uk/projects/fastqc/). Raw reads were pre-processed using Trimmomatic (version 0.36) to trim off PCR primers, sequencing adaptors, and low-quality ends (average quality lower than 20 within a 4 nucleotide (nt) window) [24]. Quality-controlled reads (average quality 20 and above, read length 40 and above) were assembled using megahit (version 1.1.3) with multiple k-mers of 21, 29, 39, 59, 79, 99, and 119 [25]. To annotate contigs taxonomically, assembled contigs were compared against the NCBI nt database (ftp://ftp.ncbi.nlm.nih.gov/blast/db/) using BLASTN (version 2.6.0+) [26]. Hits were sorted by bit scores. The top hit was defined as the hit with the maximal bit score. Only hits with e value ≤ 1 × 10^−5^ and bit score ≥ 100 were kept for contig taxonomy annotation. The naïve best-hit method was used to obtain the specific taxa assignment per contig after manual inspection of the alignments. The LCA (lowest common ancestor) algorithm (contig mode) based on multiple BLAST hits was also applied to obtain a more accurate and general taxa assignment [27]. To quantify the abundance of contigs, quality-controlled reads were mapped back to the assembled genomes using BWA-MEM (version 0.7.17) [28]. Mapped reads were quantified using samtools idxstats (version 1.5) [29]. Unmapped reads were extracted again and aligned to an in-house database containing genomes using Bowtie 2 (parameters: -a --very-sensitive --no-mixed --no-discordant -X 1000). Mapped reads and mapped bases per viral genome were calculated using bedtools. Viral genomes with at least five mapped reads were reported using R markdown (http://rmarkdown.rstudio.com/). Additionally, de novo assembled contigs were further investigated in the metagenomic pipeline of SeqMan Ultra software (Lasergene, DNAStar, USA), viral sequences were analyzed with NCBIs ORF finder (https://www.ncbi.nlm.nih.gov/orffinder/), and phylogenetic trees were built in MEGA X [30]. MUSCLE was used for multiple sequence alignment of the amino acid (aa) sequences, and the maximum likelihood method with 1000 bootstraps replicates was employed to build up phylogenetic trees. Statistical analysis was performed in IBM SPSS software version 24 using Fisher’s exact test (*p* value ≤ 0.05).

The raw sequence reads generated in this study are available at the NCBI sequence read archive (SRA) database under Bioproject accession PRJNA638077. Virus genomes generated have been deposited in GenBank under the accession numbers MT577804, MT577805, and MT591567.

## 3. Results

In total, 125 million sequencing reads were generated from 15 pools (3.520 × 10^6^ sequencing reads per pool) and applied to reference alignment and de novo analysis to construct contigs. In each mosquito pool, between 1.9% and 36.1% of the total generated sequences were classified as viral reads (Table 2). From a total number of 30,754 contigs, identification of obtained viral contigs using the blastn algorithms resulted in detection of 167 viral contigs belonging to the viral families Flaviviridae, Rhabdoviridae, Iflaviridae, Orthomyxoviridae, Dicistroviridae, Tymoviridae, Genomoviridae, and several unclassified viral taxa. The majority of the detected viral sequences were classified as ISVs. Importantly, no human pathogenic viruses were detected.

### 3.1. Mosquito-Associated Viruses

**Figure 2:**
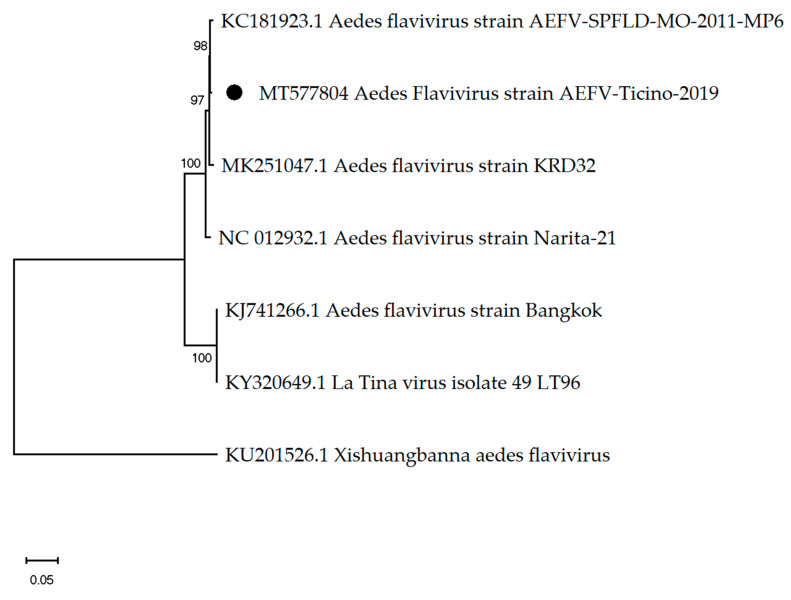
The phylogenetic tree of Aedes flaviviruses constructed using the maximum likelihood method and Kimura 2-parameter model. The percentage of replicate trees in which the associated taxa clustered together in the bootstrap test (1000 replicates) are shown next to the branches. The black dot indicates the genome sequenced in this study.

In all 15 pools, viral reads of flavivirus or flavivirus-related virus have been detected. In 14 pools, between 0.0020% and 0.5% (Table 2) of the total generated reads were assembled to Aedes flavivirus (AEFV). In the pool MO2, with 42,862 sequencing reads (0.5% of total reads), the complete AEFV of 11,038 nt in length encoding a polyprotein of 3341 amino acids in length was assembled and showed 99.62% similarity to the Aedes flavivirus strain AEFV-SPFLD-MO-2011-MP6 (GenBank accession: AGJ91136.1) (Figure 2) [31]. Additionally, in nine pools contigs with an identity above 99% to Aedes albopictus cell fusing agent virus (CFAV) have been assembled (GenBank acc: AF411835.2) [32]. 

**Figure 3:**
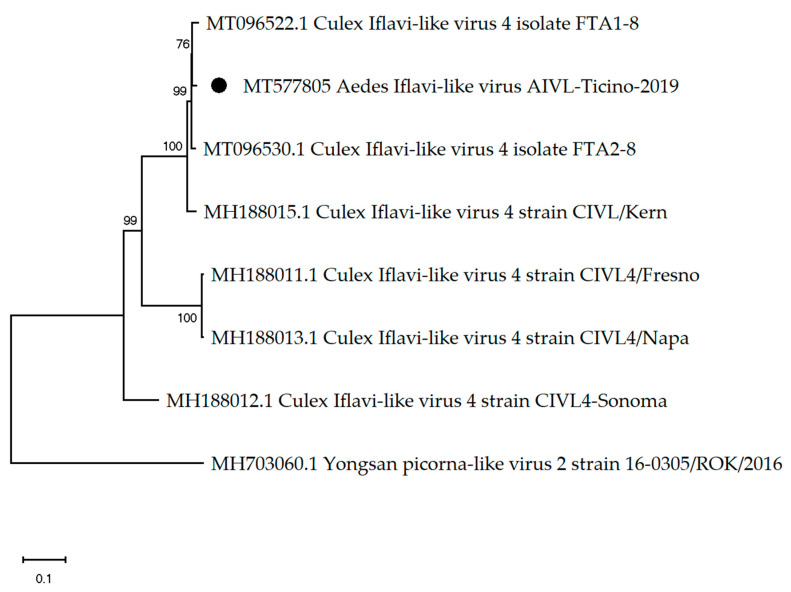
The phylogenetic tree of Aedes iflavi-like virus constructed using the maximum likelihood method and Kimura 2-parameter model. The percentage of replicate trees in which the associated taxa clustered together in the bootstrap test (1000 replicates) are shown next to the branches. The black dot indicates the genome sequenced in this study.

Interestingly, in pool MO15, the only pool with male mosquitos, a contig from the order Picornavirales with 9666 nt in length has been constructed using 165,192 sequencing reads (4.6% of total reads) (Table 2). Further analysis revealed a 9666 nt genome with 97.47% nt similarity to Culex Iflavi-like virus 4 isolate FTA18 (GenBank acc: MT096522.1) [33] (Figure 3), with a polyprotein starting at nt 298 and ending at nt 9639 (9342 nt and 3113 aa in length). The detection of Aedes iflavi-like virus in a pool of male mosquitos only was statistically significant (*p* value ≤ 0.05).

**Figure 4:**
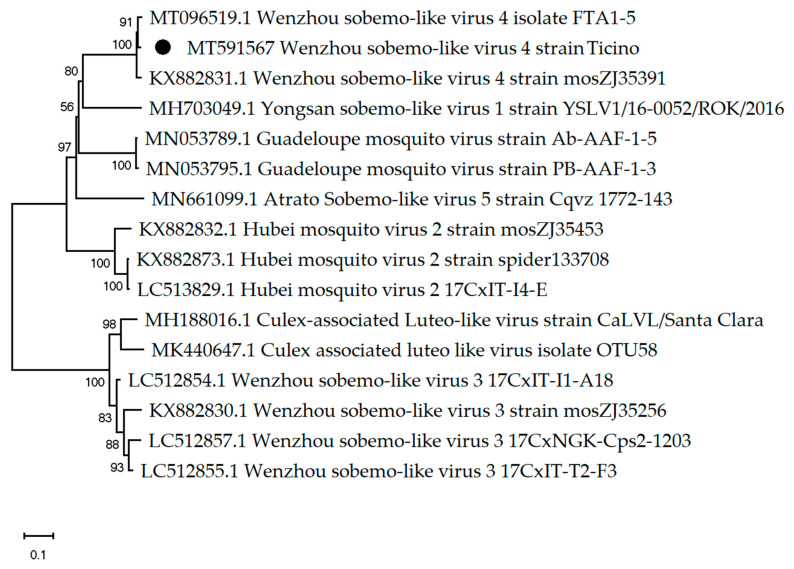
The phylogenetic tree of Wenzhou sobemo-like virus constructed using Table 2. parameter model. The percentage of replicate trees in which the associated taxa clustered together in the bootstrap test (1000 replicates) are shown next to the branches. The black dot indicates the genome sequenced in this study.

Between 1.3 × 10^5^ and 3.6 × 10^6^ sequenced reads (1.333–0.1% of the total reads) (Table 2) have been assembled to Wenzhou sobemo-like virus, which was the most abundant virus detected in all 15 pools. The metagenomic pipeline of SeqMan Ngen revealed in pool MO1 a genome of 2959 nt length and 98% nt identity to Wenzhou sobemo-like virus isolate FTA 15 (GenBank acc: MT096519.1) [33] (Figure 4). A hypothetical protein 1 of 1770 nt/589 aa in length starts at nt 67 and ends at nt 1836, and a hypothetical protein 2 of 1326 nt/441 aa in length starts at nt 1578 and ends at nt 2903. Additionally, in nine pools, between 0.037% and 1.3% of the total reads generated (Table 2) were assembled to Hubei mosquito virus 2 (GenBank acc: KX882764.1), which has previously been detected in mosquitos collected in China.

Two contigs belonging to Rebdoviridae, particularly Arboretum virus, were detected in pools MO8 and MO10. Contigs of 1064 nt and 1235 nt in length, generated from 144 and 125 sequenced reads, respectively, showed 79.6% nt identity to novel rhabdoviruses isolated from mosquitos in Peru (GenBank acc: KC994644.1) [34]. One contig of 471 nt generated from 17 sequenced reads belonging to Orthomyoviridae has shown 75.4% nt identity to Whidbey virus strain UW1 pb1 gene (GenBank acc: KX898491.1) identified in *Aedes dorsalis* in Washington. Moreover, in seven pools, sequencing reads of two unclassified viruses were assembled with high nt identity to viruses detected in Brazilian mosquitos. In seven pools, 25,294 reads with 99–100% nt identity to the putative glycoprotein gene of Guato virus (GenBank acc: KT966486.1) have been assembled [35]. In two pools, 23 and 15 reads matched in 99% of the nt to a putative glycoprotein of Kaiowa virus (GenBank acc: KT966481.1 and MF344590.1) [35].

### 3.2. Other Insect-Associated Viruses

In pool MO4, three contigs with lengths ranging from 307 to 794 nt showed 93.3%–99% nt similarity to members of the family Dicistroviridae, in particular to Aphid lethal paralysis virus isolate ALPV-CE (GenBank acc: MK704471.1), and Aphid lethal paralysis virus isolate ALPV-An (GenBank acc: JX480861.1) identified in *Aphis nerii* and several other insects species [36]. That was the only pool in which sequences assembled to Aphid lethal paralysis virus have been detected.

### 3.3. Other Viruses

Two contigs of 526 and 839 nt length showing 81% and 90% nt identity, respectively, to Lake Sarah–associated circular virus-48 were generated in pool MO12. This virus belongs to the CRESS DNA viruses identified from Lake Sarah in New Zealand (GenBank acc: KP153505.1) [37]. Viral reads assembled to a plant virus, specifically Fig fleck-associated virus from the family Tymovirdae, were detected in pools MO10 and MO11 (824 and 518 respectively) (GenBank acc: NC_015229). In pool MO13, 0.003% of the total reads have been assembled to plant associated genomovirus 3, a virus from the Genomoviridae family (GenBank acc: MH939439.1).

### 3.4. Wolbachia Sp. Detection

Finally, since only a fraction of the reads was found to have a viral origin (1.9% to 36.1%), we also investigated the presence of other infectious agents, Wolbachia in particular. Indeed, between 290 and 14,763 reads were assembled to Wolbachia wAlbB in each pool, thereby confirming that *Ae. albopictus* is naturally infected with Wolbachia strains.

## 4. Discussion

It is well known that arthropods constitute a major reservoir for many different viruses and therefore play an important role in virus spread and evolution [38,39]. High species diversity, worldwide distribution, and dense population support viral transmission and increase the risk of emerging and remerging viral diseases. Some mosquitos, including *Ae. albopictus* and *Ae. Aegypti,* are vectors for several medically important viruses such as ZIKAV, DENV, CHIKV, and WNV [40,41,42,43,44,45]. RNA viruses are particularly predisposed to causing new emerging diseases due to their inherently high mutation rate, which facilitates adaptation to new hosts [46,47]. Therefore, surveying the virome of invading mosquitos, in particular of species that are known vectors of human viruses, is of utmost importance in order to (i) detect potential spots of disease outbreaks and (ii) understand the role of mosquitos in virus evolution and spread.

In the current study, we used viral metagenomic sequencing to determine the virome of *Ae. albopictus* collected in Southern Switzerland. Importantly, genomes of medically relevant viruses were not detected. The most abundant virus genomes detected were Wenzhou sobemo-like virus and AEFV. Wenzhou sobemo-like virus has previously been found in mosquitos [48], but plants can also serve as natural host of sobemoviruses [49]. AEFV is an ISFV which replicates in mosquito cells but is unable to replicate in mammalian cells [50,51,52]. While to our knowledge this is the first report of fully sequenced AEFV in Switzerland, the virus has previously been detected in *Ae. albopictus* collected in Northern Italy, with a prevalence of 77.5% in 2008 and 16.8% in 2012 [53,54].

It would be interesting to investigate whether a high prevalence of ISFVs such as AEFV in Southern Switzerland (this study) and Northern Italy [53,54] correlates with a low prevalence of other medically important viruses. Indeed, cell fusing agent virus (CFAV) and *Culex* flavivirus, two other members of the ISFVs, are known to inhibit WNV, ZIKV, and DENV [15,55,56]. On the other hand, medically important arthropod-borne flaviviruses may have evolved from ISFVs [57,58].

Not only viruses have been shown to inhibit the replication of other viruses. *Wolbachia* sp. is an intracellular endosymbiont that reduces the ability of *Ae. aegypti* to transmit ZIKV and DENV by restricting their replication [59]. Indeed, *Wolbachia*-infected *Ae. aegypti* mosquitos have been released into the environment in Australia with the aim to supress the spread of DENV [17]. *Wolbachia* reads have been detected in all our mosquito pools. This was expected, as previous studies revealed a prevalence of *Wolbachia* in *Ae. albopictus* of >95% [60,61,62].

An interesting observation of this study was that Aedes iflavi-like virus genomes were found exclusively in a pool of male mosquitos. The most closely related viruses have been detected in mosquitos collected in Spain and California [33,63]. Considering that most of the studies have been performed with female mosquitos, and that the iflavi-like virus has been found in a pool of female *Culex* spp. in California, there is no explanation why in the present study iflavi-like virus has been detected only in a pool of male *Ae. albopictus*. However, the number of mosquitos collected at different locations varies greatly, and the presence of the virus may be location-specific. While it is rather unlikely that only a single male mosquito in the pool hosted the virus when considering the large number of assembled reads, this possibility cannot be excluded. The transmission of ISVs is not fully understood yet, and the transmission occurs mainly vertically from the female to progeny or on the breeding sites [33,64]. Further investigations would be necessary to determine whether this virus is sex- or location-specific.

In conclusion, this study presents a snapshot of the virome of established Swiss populations of *Ae. albopictus* and sets the basis for future metagenomic analyses to explore the spatial and temporal dynamics of the virus diversity in these mosquitos. This kind of study may also contribute to a better understanding of virus–virus interactions and thereby support novel strategies to prevent arbovirus diseases. The world of the ISFVs and ISVs remains largely unexplored but, if better known, may yield important insights into viral evolution and the role of these viruses in the emergence and transmission of pathogenic viruses.

## Figures and Tables

**Table 1:** Summary of the mosquito samples used in this study. Male mosquitos were collected at all locations and during the whole sampling period.

Pool Name	Sample Location	Position	Number of Mosquitos	Gender	Sampling Time Point
MO1	Lugaggia	46.062 N 8.969 E	5	female	August
MO2	Manno	46.034 N 8.918 E	16	female	August
MO3	Lugano1	46.028 N 8.964 E	42	female	August
MO4	Porza	46.029 N 8.960 E	21	female	August
MO5	Lugano1	46.028 N 8.964 E	48	female	September
MO6	Lugano1		34	female	September
MO7	Lugano1		35	female	September
MO8	Lugano1		40	female	September
MO9	Muzzano	45.996 N 8.911 E	40	female	September
MO10	Muzzano		40	female	September
MO11	Muzzano		40	female	September
MO12	Muzzano		50	female	September
MO13	Lugano2	45.997 N 8.944 E	59	female	September
MO14	Lugano2		28	female	September
MO15	Mix		40	male	mix

**Table 2:** Viral reads detected shown as a percentage of the total number of reads generated from each pool of mosquitos.

Virus	MO1	MO2	MO3	MO4	MO5	MO6	MO7	MO8	MO9	MO10	MO11	MO12	MO13	MO14	MO15
Aedes albopictus cell fusing agent virus	0.003%	0.0002%	-	-	-	0.0002%	0.0003%	0.0001%	0.0006%	0.0001%	0.0002%	0.0002%	-	-	-
Aedes flavivirus	-	0.5%	0.06%	0.005%	0.03%	0.06%	0.01%	0.02%	0.007%	0.04%	0.02%	0.04%	0.04%	0.002%	0.153%
Aphid lethal paralysis virus	-	-	-	0.001%	-	-	-	-	-	-	-	-	-	-	-
Arboretum virus	-	-	-	-	-	-	-	0.002%	-	0.001%	-	-	-	-	-
Culex Iflavi-like virus 4	-	-	-	-	-	-	-	-	-	-	-	-	-	-	4.6%
Fig fleck-associated virus	-	-	-	-	-	-	-	-	-	0.004%	0.008%	-	-	-	-
Guato virus	-	0.002%	-	0.0004%	-	0.0003%	0.001%	-	-	0.001%	0.002%	0.0003%	-	-	-
Hubei mosquito virus 2	1.3%	0.058%	-	0.42%	0.77%	0.19%	0.3%	0.2%	-	0.037%	-	0.2%	1.2%	-	-
Kaiowa virus	-	0.0002%	-	-	-	0.0002%	-	-	-	-	-	-	-	-	-
Lake Sarah-associated circular virus-48	-	-	-	-	-	-	-	-	-	-	-	0.002%	-	-	-
Plant associated genomovirus 3	-	-	-	-	-	-	-	-	-	-	-	-	0.003%	-	-
Wenzhou sobemo-like virus 4	21.4%	1.3%	33.1%	19.9%	29.0%	13.8%	17.9%	15.7%	20.5%	17.3%	12.6%	15.5%	25.2%	14.4%	31.4%
Whidbey virus	-	0.0002%	-	-	-	-	-	-	-	-	-	-	-	-	-
Total % of viruses	22.7%	1.9%	33.2%	20.3%	29.8%	14.0%	18.2%	16.0%	20.5%	17.4%	12.6%	15.7%	26.5%	14.4%	36.1%
Reads generated in million	4.2	9.4	6.7	7.5	7.1	9.9	12.2	9.5	8.1	20.6	6.7	8.9	6.8	3.5	3.6
